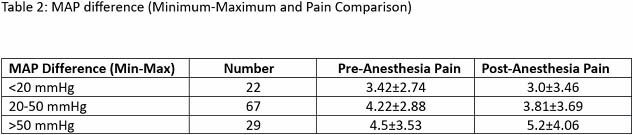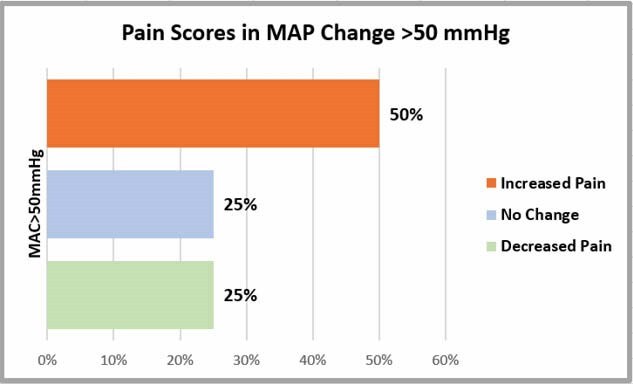# 929 Evaluating Medication Number and Effect Administered During Monitored Anesthesia Care for Burn Dressing Change

**DOI:** 10.1093/jbcr/iraf019.460

**Published:** 2025-04-01

**Authors:** Fatma Ulusan Sayali, Dhaval Bhavsar, Duncan Nickerson, Julia Slater, Katherine Golson, Niaman Nazir, Anthony Kovac

**Affiliations:** University of Kansas Medical Center; The University of Kansas Health System Burnett Burn Center; Kansas University Medical Center; The University of Kansas Health System Burnett Burn Center; University of Kansas Health System, Burnett Burn Unit; University of Kansas Medical Center; University of Kansas Medical Center

## Abstract

**Introduction:**

Under monitored anesthesia care (MAC), patients breathe spontaneously under moderate to deep sedation accompanied by various levels of analgesia. MAC has safely been used for burn dressing changes (BDC). Dosing, respiratory and hemodynamic effects of various medications used during MAC for BDC has not been completely evaluated.

**Methods:**

This prospective observational study approved by our IRB was conducted from May 15, 2023, to July 25, 2024. After informed consent, 80 patients ≥18 years old were enrolled. A total of 118 MACs were evaluated regarding patient demographics, MAC duration, instances of apnea, hypoxia, and hypotension. Statistics was evaluated by T-test and ANOVA. P< 0.05 was set as a statistical significance. Choice and timing of anesthetics, doses, hypotension, changes in mean arterial pressure (MAP), apnea, hypoxia, and pre- vs post- MAC pain scores (0=no pain, 10=maximum pain) were evaluated.

**Results:**

Of 80 patients, most were Caucasian males with BMI >30, cardiac comorbidities, TSBA >20% with 69% having 3rd degree burns. MAC duration varied from 25 to 150 minutes. Of 118 MACs, apnea was observed in 84 cases, hypoxia in 34, and hypotension in 24. Most commonly used medications were propofol, midazolam. and ketamine. Bolus administration of hydromorphone resulted in a decrease of ≥10 mmHg MAP within 10 minutes. Ketamine was used as a sub-anesthetic dose (mean 0.320 mg/kg ±0.19). Difference in MAP values and pain comparison are shown in Table 1. Among cases where the MAP maximum-minimum difference was >50 mmHg, pre-vs post anesthesia pain comparison showed a clinical but not statistically significant increase in pain scores (Graph 1).

**Conclusions:**

Multiple medications, dosing and order selection were administered. A greater number of medications administered did not necessarily imply a better MAC. When respiratory, hemodynamic and pain score changes are taken into consideration, further prospective studies are needed to help determine a recommended approach regarding MAC for BDC, preferably using a smaller number of medications enhancing a long-acting analgesia coverage.

**Applicability of Research to Practice:**

Lack of a recommended approach resulted in the administration of numerous medications and doses. The goal of proper selection and dosing is good anesthesia with minimal hemodynamic and respiratory changes, and good post-MAC pain control.

**Funding for the Study:**

N/A